# Estimating the Delay between Host Infection and Disease (Incubation Period) and Assessing Its Significance to the Epidemiology of Plant Diseases

**DOI:** 10.1371/journal.pone.0086568

**Published:** 2014-01-22

**Authors:** Melen Leclerc, Thierry Doré, Christopher A. Gilligan, Philippe Lucas, João A. N. Filipe

**Affiliations:** 1 UMR 1349 Institute for Genetics Environment and Plant Protection, Institut National de la Recherche Agronomique – Agrocampus Ouest – Université Rennes 1, Le Rheu, France; 2 UR 546 Biostatistics and Spatial Processes Unit, Institut National de la Recherche Agronomique, Avignon, France; 3 UAR 1240 Unité Impacts Ecologiques des Innovations en Production Végétale, Institut National de la Recherche Agronomique, Thiverval-Grignon, France; 4 UMR 211 Agronomie, AgroParisTech, Thiverval-Grignon, France; 5 UMR 211 Agronomie, Institut National de la Recherche Agronomique, Thiverval-Grignon, France; 6 Epidemiology and Modelling Group, Department of Plant Sciences, University of Cambridge, Cambridge, United Kingdom; The University of Tokyo, Japan

## Abstract

Knowledge of the incubation period of infectious diseases (time between host infection and expression of disease symptoms) is crucial to our epidemiological understanding and the design of appropriate prevention and control policies. Plant diseases cause substantial damage to agricultural and arboricultural systems, but there is still very little information about how the incubation period varies within host populations. In this paper, we focus on the incubation period of soilborne plant pathogens, which are difficult to detect as they spread and infect the hosts underground and above-ground symptoms occur considerably later. We conducted experiments on *Rhizoctonia solani* in sugar beet, as an example patho-system, and used modelling approaches to estimate the incubation period distribution and demonstrate the impact of differing estimations on our epidemiological understanding of plant diseases. We present measurements of the incubation period obtained in field conditions, fit alternative probability models to the data, and show that the incubation period distribution changes with host age. By simulating spatially-explicit epidemiological models with different incubation-period distributions, we study the conditions for a significant time lag between epidemics of cryptic infection and the associated epidemics of symptomatic disease. We examine the sensitivity of this lag to differing distributional assumptions about the incubation period (i.e. exponential *versus* Gamma). We demonstrate that accurate information about the incubation period distribution of a pathosystem can be critical in assessing the true scale of pathogen invasion behind early disease symptoms in the field; likewise, it can be central to model-based prediction of epidemic risk and evaluation of disease management strategies. Our results highlight that reliance on observation of disease symptoms can cause significant delay in detection of soil-borne pathogen epidemics and mislead practitioners and epidemiologists about the timing, extent, and viability of disease control measures for limiting economic loss.

## Introduction

Invasions of semi-natural systems by plant pathogens can cause substantial economic and ecological damage [1], [2], [3], [4], [5]. Invasions of soilborne plant pathogens, however, have received less attention than their airborne counterparts [6]. Unlike airborne pathogens, which can disperse over very large distances, soilborne plant pathogens generally disperse over short distances and invade host plant populations on smaller spatial scales [7], [8], [9], [10]. However, inoculum stages of these pathogens can be carried over considerable distances, through water, animal movement, and human agricultural and trade practices [11], and survive in the soil from season to season [12]. Therefore, large outbreaks (epiphytotics) of soilborne plant diseases can occur and cause severe crop losses [5], [13], [14], [15]. However, as plant soilborne pathogens infect and spread cryptically underground much before the emergence of visible disease symptoms [7], it is difficult to assess disease risk and prevent, detect, and control the development of epidemics without resorting to pre-emptive treatments harmful to soil ecosystems and the general environment [12]. In order to design and target appropriate disease management strategies, it is crucial, therefore, to know the incubation period (from host infection to expression of disease symptoms) associated with a given pathogen and host. Perhaps not surprisingly, there is very limited information about the expected magnitude and between-individual variability of the incubation periods of soilborne plant pathogens [16]. In this paper, we present the results of experiments for measuring the incubation period of the ubiquitous soilborne pathogenic fungus *Rhizoctonia solani* [17] in sugar beet. We identify different probability distribution models that fit the observations, and assess the epidemiological implications of making different assumptions about the incubation period, by studying pathogen and disease spread in a spatially-explicit epidemiological model.

**Figure 1:**
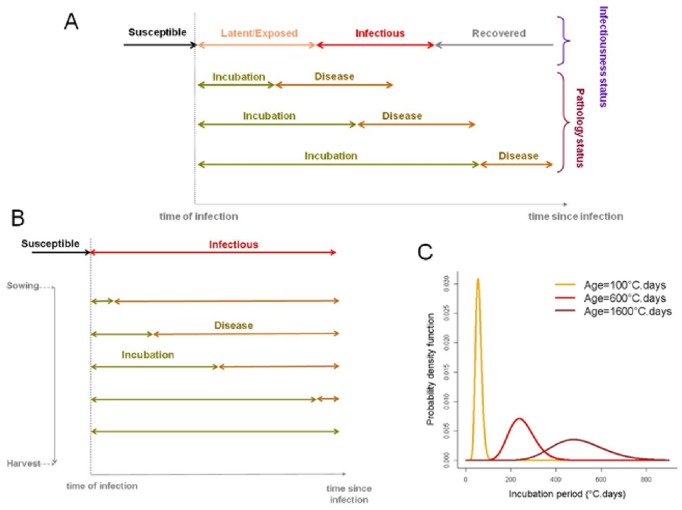
Epidemiological, within-host, life-cycle periods of a pathogen. A) An infected host may exhibit differing combinations of infectiousness state (*susceptible-latent-infectious-recovered*) and pathology state (*incubation-diseased*), depending on characteristics and conditions of the host and pathogen. B) In the case of a soilborne plant disease, the appearance of visible disease symptoms can be delayed when infection occurs late in the crop season and/or the host is mature. C) Illustration of an age-varying distribution of the incubation period in the *R. solani* – sugar beet pathosystem.

**Figure 2:**
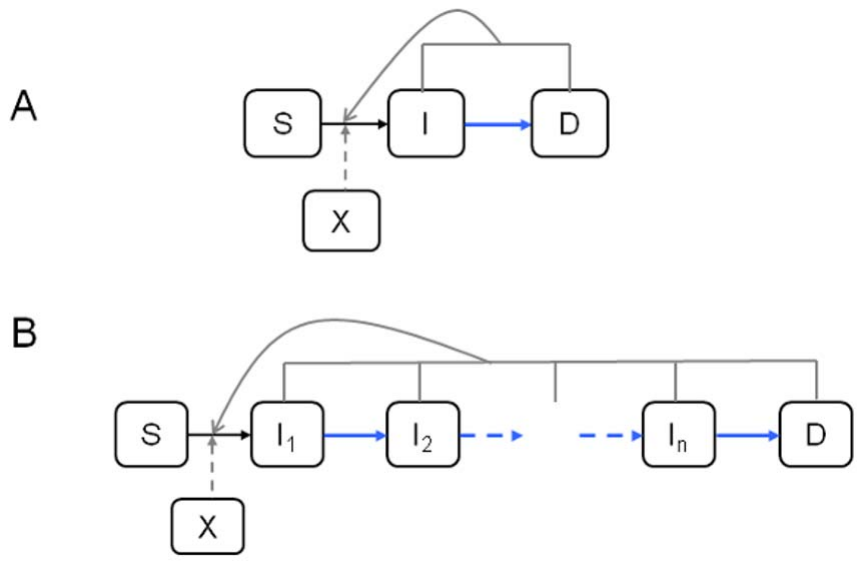
Epidemiological models. Compartmental structure (Susceptible – Infectious - Diseased or symptomatic) and dynamic transitions of each individual in the host population. A) The SID model has an exponentially-distributed incubation period. B) The S-I(n)-D model, where the infectious compartment is subdivided into n compartments prior to appearance of disease symptoms, has an Erlang-distributed incubation period (sum of n exponentially- distributed random variables). In this paper, the infectious and diseased states of one host are equally infectious and contribute to pathogen spread to other hosts, and, participate to their transition from state S to state I (grey lines). Primary infection is determined by amount of resident inoculum (X) near a susceptible host, and also contributed to its infection (broken grey lines).

The epidemiology of transmissible diseases is characterised by the *infectiousness* status of the individual hosts exposed to a given pathogen; but this status is usually unobserved (hidden) and not easily determined microbiologically [18], [19]. Therefore, health and disease management are generally informed by the *pathology* status of the hosts, whether human, animal, or botanical [20]. The relative development of the two statuses in infected hosts can be disparate (Fig. 1A) depending on the infectious agent and host species, with implications to the feasibility of controlling disease outbreaks [21]. Characterising the incubation period of a pathogen-host system relies on the ability to determine and relate the relative development of host infectiousness and pathology, which is limited by challenges in collecting appropriate data [22]. Here, we are interested in modelling plant disease outbreaks, and, in particular, in characterising the incubation period of soilborne pathogens in host plants by relating their infectiousness and pathology (Fig. 1B). Epidemiological models of disease spread in human, animal [23], [24] or plant [16], [25] populations typically rely on a compartmentalisation of the infectiousness status as *Susceptible*, *Exposed* (or *Latent*), *Infectious*, and *Removed* (or *Recovered*) classes, or subsets or extensions thereof depending on the specific pathogen life-history and host species. In order to merge infectiousness and pathology statuses, a *Diseased* state is usually added. For plant pathogens, disease expression usually occurs in already infectious hosts and does not stop host from being infectious (Fig. 2A) [26]; in this context, state I represents infectious incubation. The simplest compartmental models implicitly assume the between-host distribution of the incubation period (in fact, the residence time in any of the compartment states) is a negative exponential. As this convenient assumption can be biologically implausible, adaptations of the compartmental modelling framework have been proposed that have more flexible residence-time distributions with non-zero mode (Fig. 1C), such as Gamma or Erlang probability density functions [27], [28], [29]. For soilborne plant pathogens, for which a SID epidemiological model is often appropriate (Fig. 2A) [8], [30] we implement the adaptation by dividing the non-symptomatic infectious stage into multiple states (Fig. 2B). Several modelling studies have examined the epidemiological consequences of differing assumptions about latent and infectious period distributions [31], [32], [33], but few works have studied the incubation period and associated disease management implications, or used empirical data.

After presenting our observations of the incubation period of *R. solani* in sugar beet, we fit suitable probability models to their distribution, and demonstrate its dependency on host age. Furthermore, we study the conditions for a significant lag between observable disease epidemics and cryptic pathogen epidemics across a host population, and the sensitivity of this lag on differing modelling assumptions about the incubation period. We address these questions by developing and exploring an epidemiological model of pathogen spatial spread that incorporates the data-fitted distributions.

## Materials and Methods

### Pathosystem

In this study we considered the saprotrophic fungus *Rhizoctonia solani* anastomosis group (AG) 2-2 IIIB (isolate G6) which parasitizes various plant crops, such as sugar beet, maize, and rice. On sugar beet, this pathogen causes the economically important root rot (or crown rot) disease [34]. *Rhizoctonia solani* spreads locally from infected plants to neighbouring non-infected plants, causing the development of patches of disease during the crop-growing season. The isolate AG2-2 IIIB tends to spread late on mature plants [17]. However, as the susceptibility of sugar beet to *R. solani* does not change significantly with age, the fungus can colonize sugar beet at any stage of a crop season provided suitable environmental conditions occur. The presence of root rot disease is often shown by above-ground symptoms of crown rot, wilting, and, when epidemics start early in the growing season, pre-emergence and post-emergence damping off.

### Experimental Measurements of the Incubation Period

We inoculated the roots of sugar beet plants of different ages with *R. solani*, and measured the time between inoculation and above-ground detection of symptoms in field conditions. Experiments were carried out in the INRA experimental station at Le Rheu, France (coordinates 48°06′ N, 1°48′ W) in 2010 and 2011, with the permission of the Inra experimental unit UE787. The sugar beet crop (cv Skipper) was sown manually on April 9^th^ 2010 and on April 8^th^ 2011 and was irrigated to prevent soil dehydration and plant hydric stress. As strains of *R. solani* pathogenic to sugar beet had not been introduced and sugar beet had not been grown previously in these plots, we assumed that the soil was free of inoculum before the experiments. We sowed sugar beet at the vertices of a regular lattice with a 80 cm spacing; a distance large enough to prevent undesirable infections between neighbouring plants in these conditions [35].

We used infested barley seeds as inoculum of *R. solani*. First, barley seeds were soaked with water before autoclaving (2×1 h at 115°C, with a 24 h interval between autoclaving); then the autoclaved barley was inoculated with mycelial plugs removed from the margins of seven-day old colonies grown on malt agar at 20°C. Finally, the inoculated seeds were incubated for three weeks at 20°C.

In order to assess the evolution of the incubation period at different ages of plant root infection, we inoculated sugar beet at ages 14, 32, 46, 60, 74, 88, 102, 116 and 130 days after sowing. In the experimental area, individuals were randomized in 3 blocks (i.e. south, middle and north) to assess the potential effect of the position in plots on the incubation period. Inoculations consisted in placing inoculum units (three infested barley seeds) in contact with plants 3 cm below-ground. Above-ground symptoms of root rot disease were assessed visually at least every two days. We have adopted as a measure of incubation period the time interval between inoculation and detection of the first symptoms. In our statistical analyses, we ignore this censoring in the data as the censoring interval is small. For each host age, we infected at least 40 plants. Specifically, we obtained measures of the incubation period of 78, 52, 53, 49, 46, 49, 45, 46, and 46 individuals at ages 14, 32, 46, 60, 74, 88, 102, 116 and 130 days, respectively (Table 1).

**Table 1:** Experimental incubation period data by host age: Number of plants inoculated, mean, and standard deviation.

Age (days)	Age (°C.days)	Number of individuals	Mean (days)	SD (days)	Mean (°C.days)	SD (°C.days)
18	182.35	78	6.5	0.9	79.19	11.37
32	359.25	52	10.0	2.3	128.72	31.11
46	542	53	14.4	3.1	198.69	42.91
60	607.05	49	18.5	1.9	279.52	33.47
74	811.15	46	21.2	4.7	371.94	82.56
88	1053.95	49	25.4	5.2	445.88	87.98
102	1303.35	45	26.8	5.8	442.43	90.1
116	1545	46	31.8	10.2	486.92	149.35
130	1764.85	46	37.4	10.7	537.60	133.30

Two time scales are often used to measure processes in plant systems: calendar time in days, which is useful for practitioners, and time in degree-days, which incorporates temperature-dependence in plant and pathogen responses [36]. In this work, we measure time in degree-days because temperature was a key abiotic variable that was not controlled during our experiments.

### Age-specific Incubation Period Analysis

**Figure 3:**
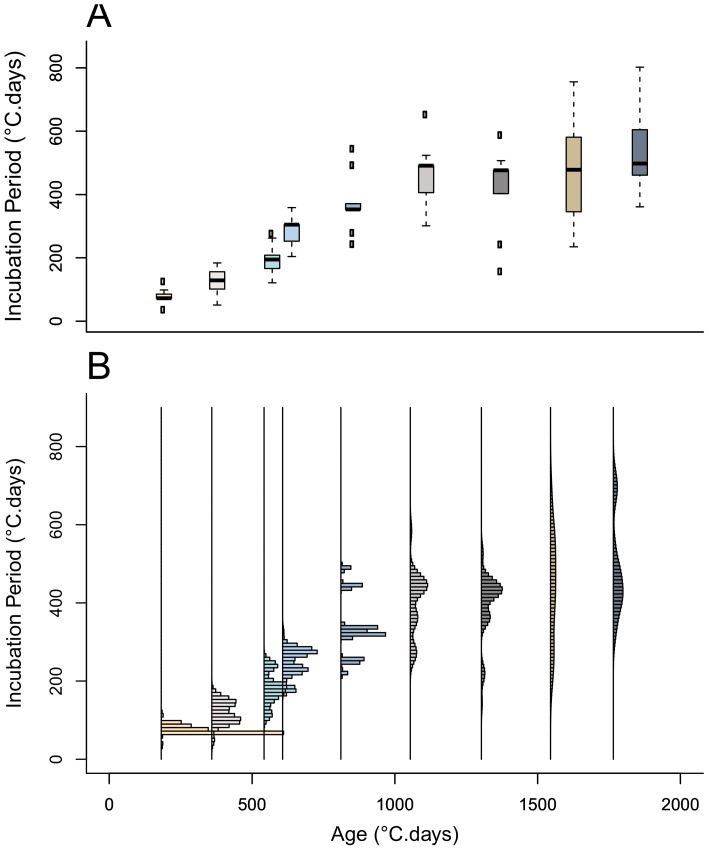
Experimental measurements of the incubation period. Experimental data on the incubation period of *R. solani* in sugar beet plants of nine differing ages. Dispersion of the incubation period within each plant age group, shown in: A) boxplots, and B) frequency distributions (histograms).

While the distribution of the incubation period of the inoculated plants exhibits more than one mode for some host ages (Fig. 3B), in this study we fitted uni-modal distribution models to the data. First, the limitations in the data may be responsible for some of the apparent multi-modality while an assumption of unimodality seems biologically plausible. Second, a uni-modal analysis is simpler to implement and offers an easily interpretable first description of the incubation period. Thirdly, in all cases (Fig. 3B), there is a dominant mode that we expect to be the main determinant of the mode in the fitted distribution model. We fitted alternative probability density function (pdf) models, Gamma, Weibull, Lognormal, and exponential, to the incubation period data allowing for mutually-independent sets of pdf parameters among host-age groups (see Appendix S2 for definition of pdfs). The parameters were evaluated using maximum-likelihood estimation and neglecting the censoring imposed by the two-day observation frequency, i.e., assuming the data represent the actual time period between infection and first emergence of symptoms for each plant. We used the Akaike information criterion (AIC) metric to compare the goodness of fit of the alternative pdf models over all age groups and for each age group. For each plant age group, we also compared the survival function associated with each two-parameter distribution, Gamma, Weibull and Lognormal, against its non-parametric Kaplan-Meier estimator [37]. We assessed the effect of the location of individuals in plots (south, middle and north) on the incubation period, by age, by using Cox proportional hazards regression. As we found this covariate to be non-significant, we did not include it in subsequent analyses. All statistical analyses were performed using the free software R [38].

### Age-varying Models of the Incubation Period Distribution

We built age-varying models of the incubation period distribution following a semi-empirical approach. First, we assume that the *incubation period* (*T*) is a time- or age-varying Gamma-distributed random variable,
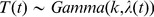
(1a)with constant shape parameter *k* and an age-dependent rate parameter *λ(t)*. Detailed analyses (see Appendix S3) suggest that the rate parameter is described by a decreasing exponential function of age *t* with non-zero asymptote *c*:
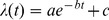
(1b)which allows the mean and variance of the incubation period to increase with age and asymptote k/c and k/c^2^, respectively. Second, we consider an Erlang distribution, i.e. a Gamma pdf with integer shape parameter [39], which offers a tractable way of conferring Gamma distributions to residence times in compartmental epidemiological models [29], [31]. The reason for this tractability is that an Erlang-distributed random variable with parameters k and λ is the sum of k exponentially-distributed random variables with rate parameter λ. Here, we consider this particular case of model (1a), i.e., T∼Erlang(k, λ(t)), and compare age-varying distribution models for the incubation period where k is a free integer parameter. Note that k = 1 corresponds to the exponential distribution.

The probability densities of the parameters k, a, b and c, were estimated from the experimental data using a Bayesian framework with a likelihood function based on (1), and non-informative prior distributions. Posterior densities were obtained via Markov Chain Monte Carlo (MCMC) parameter sampling run on OpenBUGS [40], whose outputs were analysed with R software [38] (see Appendix S3 for more details). An assessment of the adequacy of Erlang and exponential models was made by examining Box-and-Whisker plots (Boxplot) of the MCMC posterior distributions of the incubation period, rate parameter λ(t), and mean and variance associated with each of the pdf models. Finally, for assessing the relative goodness of fit of the age-varying pdf models we used the Deviance information criterion (DIC).

### Epidemiological SID Model with Differing Incubation Period Distributions

We demonstrate the importance of the assumed distribution of the incubation period, by simulating the spread of the pathogen in a host population and contrasting the dynamics of the cryptically- and symptomatically-infected parts of the population. Specifically, we simulate soilborne disease epidemics in spatially-explicit, stochastic plant population model with SID (*Susceptible-Infectious-Diseased*) compartmental structure, and with either an Erlang distribution (Fig. 2B) or the more commonly used exponential distribution (Fig. 2A) for the incubation period. For soilborne plant diseases, the host latency is often relatively short or unknown [8], so its inclusion in the model is not essential to our central question.

The dynamics of a stochastic SID model (Fig. 2A) can be modelled using a discrete-event, interacting-population Markov process [41], [42] where each individual can be in one among the states S, I, D. This process is defined in continuous time t by the probabilities of transition for each individual host in the population, conditional on its current state. We assume that the individuals are equally infectious in the I and D states. In a non-spatial (*mean-field*) model, in which the individuals interact independently of their relative location, the transition probabilities, during an infinitesimal time lag dt, are given by [43]:
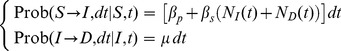
(2)where β_p_ is the rate of primary infection, β_s_ is the rate of secondary infection, μ is the rate at which infected hosts become symptomatic (after which they can be detected), and N_I_(t) and N_D_(t) are the numbers of individuals in the host population that are in states I and D, respectively, at time t. The transition probabilities (2) hold for an incubation period T that is exponentially distributed with mean 1/μ and variance 1/μ^2^, i.e., for a model with a single infectious compartment (k = 1). The extension of (2) to a non-spatial S(k)ID model (Fig. 2B) with k infectious compartments (*I_1,_…,I_k_*), i.e., a Gamma-distributed incubation period with mean λ = kμ and integer shape parameter k, is:
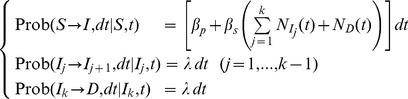
(3)


### Spatially Explicit SID Model with Differing Incubation Period Distributions

The spread of plant soilborne pathogens within a host population is often local, between nearest neighbour plants [9], [35], [44]; therefore, non-spatial models can be poor at predicting epidemics development. In an individual-based spatial model where pathogen transmission occurs between nearest-neighbour plants (i.e. within a von Neumann neighbourhood), the transition probabilities for each host (i) in the population at time t, conditional on its current state, are given by [43]:
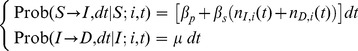
(4)for a model with a single infection compartment (k = 1), and by
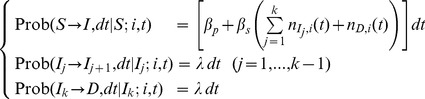
(5)for a model with k infection compartments and rate of transition from state Ii to state Ii+1 given by λ = kμ. In (4)-(5), nI,i(t) and nD,i(t) are the numbers of nearest neighbours of individual i that are in the states I and D, respectively, at time t, and nIj,i(t) is the corresponding number for the sub-state Ij.

We used the spatially-explicit models (4) and (5) to assess the impact of making different assumptions about the incubation period distribution on the epidemic dynamics; namely, the appearance of a lag between the spread of infection and the emergence of above-ground disease symptoms across the host population.

### Rates of Primary and Secondary Infection

In the case of fungal soilborne pathogens, the rate of primary infection can decline over time; it has been observed in the laboratory that there is decline in the number and efficiency of the inoculum units [9], [44]. In addition, in field conditions, the germination and growth of residuals inocula of *R. solani* is also driven by abiotic conditions such as moisture and temperature [45]. Thus, we consider that epidemics caused by *R. solani* start at a theoretical time t_0_ after sowing when environmental conditions are suitable for fungal growth. With these assumptions, the rate of primary infection β_p_ is given by a decreasing exponential function of time with a delay:
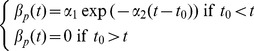
(6)


The spread of *R. solani* in crop populations with secondary infection rate β_s_ occurs predominantly between nearest neighbour plants, both in the laboratory [9], [44] and in field conditions [35]. The rate of secondary infection of soilborne fungal pathogens, and thus their ability to invade host populations, depends on several factors; for example, the distance between neighbouring host plants (i.e. spacing at sowing or planting in crop systems). In field conditions, however, there is a myriad of known biotic or abiotic factors that may affect the secondary transmission of *R. solani* and thus impact the emergence of epidemics. For simplicity, for the purpose of this paper of demonstrating the effect of incubation period distributions on epidemic development, we do not focus on a specific environmental factor, and thus consider the rate of secondary infection to be constant over time in the population model (i.e. β_s_(t) = β_s_ >0).

### Simulation of the Spatially-explicit Epidemics

In order to compare the dynamics of disease symptoms and hidden infections, we simulated stochastic epidemics in scenarios with low (β_s_ = 5e-7) or high (β_s_ = 5e-4) rate of secondary infection, and with an early (t_0_ = 0°C.days) or late (t_0_ = 700°C.days) start of the epidemic in relation to the time of crop sowing. For primary infection we used α_1_ = 0.002 and α_2_ = 0.008 based on previous parameter estimation [26]. We simulated the stochastic epidemics by running our spatially-explicit SID model on a 100 site by 100 site square lattice up to 2500°C.days. For coding the simulation we used the *direct method* algorithm [46], which gives exact realisations of Markov processes. We determined the time to emergence of disease symptoms after each new individual infection (*incubation period*), by sampling from Erlang(k,λ(t)) or Exponential(λ(t)) *pdfs* with age-varying parameters based on the means of the Bayesian posterior distributions of parameters a, b and c. As our simulation study is aimed at a demonstration of principle, it is not relevant in this case to incorporate parameter uncertainty associated with the estimated posterior distributions. These *hierarchical hidden Markov models* were implemented in C++ and the outputs were analysed with R software.

## Results

### Empirical Incubation Period Distribution

The experimental results show significant age-specific variation in the incubation period (time from inoculation to disease symptom expression) of sugar beet plants inoculated with *R. solani* (Fig. 3). There is generally an increase in the mean and variance (Table 1) and in the dominant mode and dispersion (Fig. 3) of the incubation period with increasing age of the sugar beet. The mean and variance increased up to age 1000°C.days, after which they stabilised.

### Models of the Incubation Period Distribution

**Figure 4:**
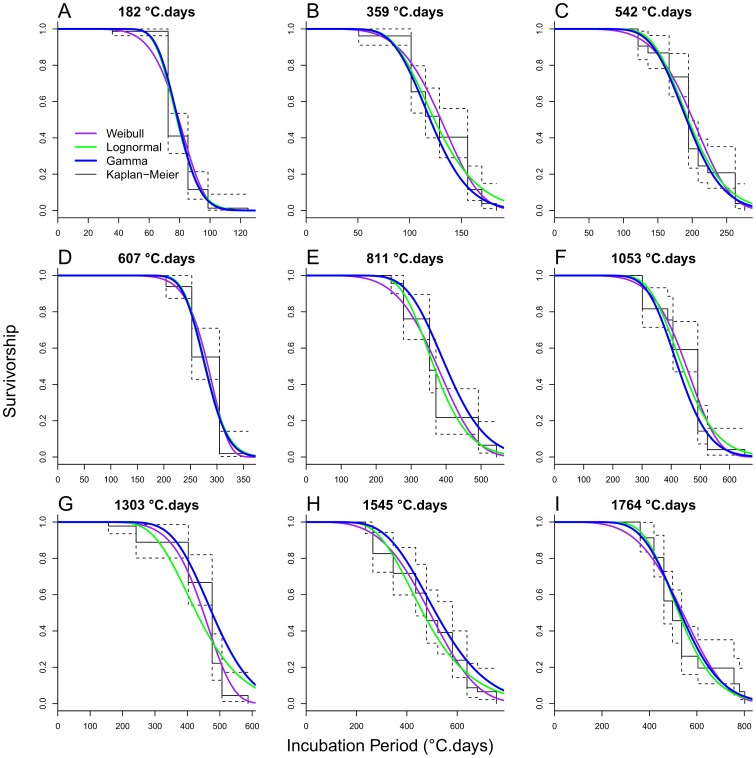
Survivorship of the distribution models fitted to the incubation period data by host age. Gamma (blue line) Lognormal (green), Weibull (purple), and estimated non-parametric Kaplan-Meier (black) survival function. Age of host plants at inoculation: (A) 18 days (182°C.days), (B) 32 days (359°C.days), (C) 46 days (542°C.days), (D) 60 days (607°C.days), (E) 74 days (811°C.days), (F) 88 days (1053°C.days), (G) 102 days (1303°C.days), (H) 116 days (1545°C.days),and (I) 130 days (1764°C.days).

For each age of the plants at inoculation, the survival functions associated with the Gamma, Lognormal, and Weibull distributions fitted to the incubation period data are close to the non-parametric Kaplan-Meier estimator of incubation survivorship (Fig. 4); hence, either of these distributions appears to be a suitable candidate model of the incubation period distribution. Based on the AIC relative goodness of fit measure, the data are best described by the Gamma distribution for plant ages 182 and 542°C.days, by the Erlang distribution for 811°C.days, by the Lognormal distribution for 1764°C.days, and by the more flexible Weibull distribution for the other plant ages (Table 2). However, the differences in AIC score among distributions are less than 5 for most age groups (Table 2). The overall AIC on model fit to all age groups (sum of age-specific scores) shows negligible difference among the Gamma (4998), Weibull (4995), and Erlang (4997) distributions, but indicates a less good fit of the Lognormal distribution (5018) (Table 2). Not surprisingly, the single-parameter exponential distribution is clearly inadequate with overall AIC 6085. These results suggest that, while the Weibull distribution emerges as the best candidate distribution model, the Gamma distribution and its special case of the Erlang distribution, which is convenient to use in compartmental models, are appropriate and may be implemented in our population models (Appendix S2). Henceforth, we focus on these distributions.

**Table 2:** Distribution models fitted to the incubation period data by host age, Gamma, Lognormal, Weibull, Erlang, and exponential: parameters and Akaike information criterion (AIC) score.

	Gamma			Weibull			Lognormal			Erlang			Exponential	
Total AIC	4998.06			4994.65			5017.56			4996.8			6084.6	
Age in days(°C.days)	AIC(*)	shape	rate	AIC(*)	shape	rate	AIC(*)	Meanlog(**)	sdlog(**)	AIC(*)	shape	rate	AIC	rate
18 (182.35)	**604.40**	47.88	0.600	620.73	6.47	0.0119	**607.52**	4.36	0.15	**604.50**	48	0.610	840.01	0.013
32 (359.25)	**512.60**	15.30	0.120	**507.04**	4.83	0.0071	517.00	4.82	0.27	**513.00**	15	0.120	611.20	0.008
46 (542.00)	**552.50**	20.84	0.100	**552.70**	5.18	0.0046	**554.21**	5.27	0.22	**552.55**	21	0.110	669.00	0.005
60 (607.05)	**488.00**	67.72	0.240	**484.18**	9.90	0.0034	489.35	5.63	0.12	**488.05**	68	0.240	652.04	0.004
74 (811.15)	**536.11**	21.68	0.060	542.85	4.74	0.0025	**535.20**	5.90	0.21	**535.11**	22	0.060	638.52	0.003
88 (1053.95)	**582.90**	24.39	0.050	**580.60**	5.78	0.0021	**584.89**	6.10	0.21	**583.00**	24	0.050	697.81	0.002
102 (1303.35)	548.21	18.00	0.040	**527.12**	6.83	0.0021	555.82	6.06	0.26	548.24	18	0.040	640.31	0.002
116 (1545.00)	**595.53**	9.83	0.020	**592.74**	3.76	0.0018	597.83	6.14	0.33	**595.54**	10	0.020	663.30	0.002
130 (1764.85)	**577.81**	19.19	0.030	586.69	4.19	0.0017	**575.74**	6.26	0.23	**576.81**	18	0.030	672.41	0.002

(*)Bold indicates lowest or within 5 units from the lowest AIC score.

(**)Mean and standard deviation of the log transformed variable.

### Age-varying Models of the Incubation Period Distribution

**Figure 5:**
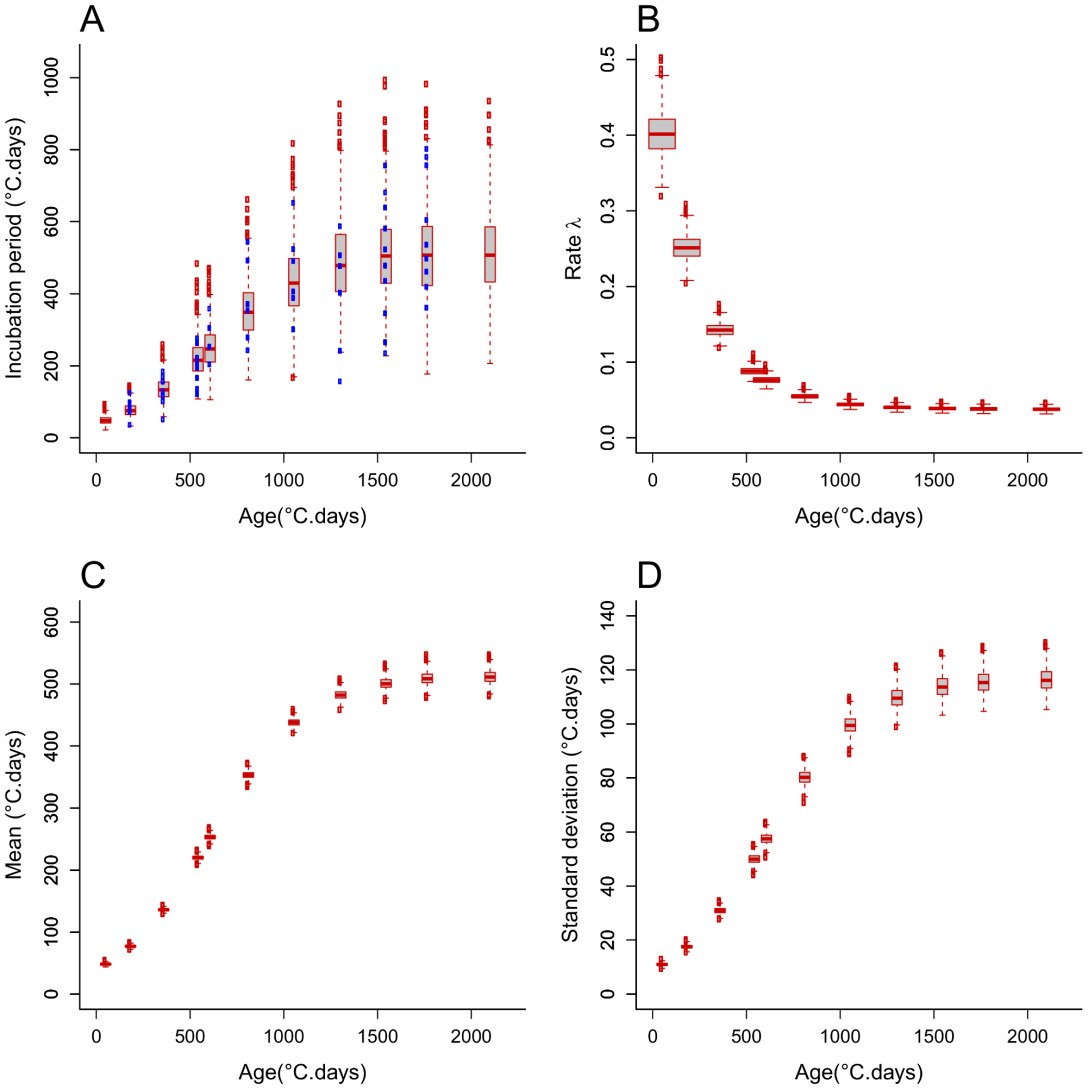
Age-varying Erlang distribution model of the incubation period of *R. solani* in sugar beet plants. MCMC posteriori Erlang distribution (A), and uncertainty in its rate parameter function of plant age λ(t) = a*exp(−b*t)+c (B), mean (C) and standard deviation (D). The posterior distributions of the Markov Chain Monte Carlo sample are shown with boxplots, where the end of dashed lines represents minimum (bottom) and maximum (top) quartiles that exclude outliers (empty circles). The shape parameter k has mode value 19. The model was fitted simultaneously to data on plants inoculated at ages 182, 359, 542, 607, 811, 1053, 1303, 1545 and 1764°C.days. Observed incubation periods are represented by blue full points in (A).

**Figure 6:**
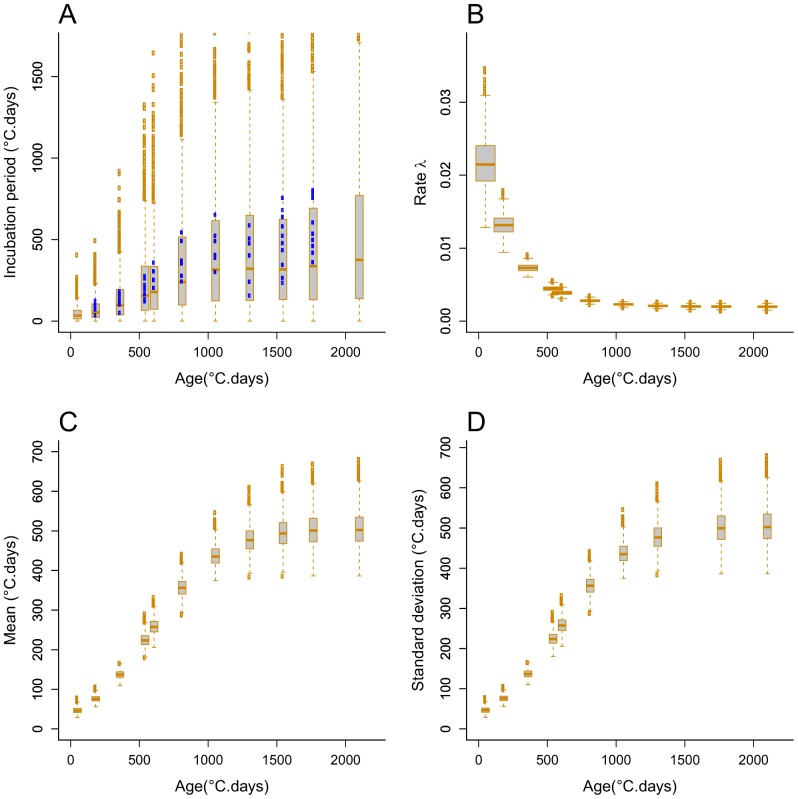
Age-varying exponential distribution model of the incubation period of *R. solani* in sugar beet plants. Similar to Figure 5, except that the shape parameter has fixed value k = 1.

We fitted alternative single-distribution models, Gamma, Erlang, and exponential, simultaneously to the incubation period data from every plant age group by allowing the rate parameter to vary with plant age *t* according to (1b). We used Bayesian MCMC parameter sampling to fit the models. According to the DIC relative goodness of fit measure (Table 3), the model with best fit is the Erlang distribution (DIC = 5071), followed closely by the Gamma distribution (DIC = 5075). The exponential distribution model fitted the data poorly (DIC = 6075). Based on the mode of the parameter’s marginal posterior distributions (Table 3), the fitted Erlang distribution has shape and rate parameters k = 19 and λ(t) = 0.450*exp(−0.00403*t)+0.0381, and the fitted exponential distribution, for which k = 1 by definition, has rate parameter λ(t) = 0.025*exp(−0.00424*t)+0.002. The posterior distributions of parameters a, b, and c are relatively tighter (narrower 95% confidence range) for the Erlang and Gamma models than for the exponential model (Table 3). Likewise, the posterior distribution of the rate parameter λ(t) shows relatively lower uncertainty (lower standard errors) for the Erlang model (Fig. 5B) than for the exponential model (Fig. 6B), especially for young plants. The mean and standard deviation increased with plant age following a sigmoid shape with asymptotes 513 and 118, respectively, for the Erlang distribution model (Fig. 5C & 5D); and with asymptotes 500 and 500, respectively, for the exponential distribution model (Fig. 6C & 6D). In line with these results, the *a posteriori* Erlang incubation period distribution (Fig. 5A), obtained via re-sampling from the fitted Erlang distribution model, matches reasonably well the median and dispersion in the experimental data (Fig. 3). The corresponding posterior distribution for the exponential distribution model (Fig. 5A) shows over-dispersion in relation to the data (Fig. 3).

**Table 3:** Age-varying incubation period distribution models fitted to experimental data on all host-age groups, Gamma, Erlang, and Exponential (k = 1): shape parameter k, parameters a, b, c in the rate parameter function of host age λ(t) given by (1b), and Deviance information criterion (DIC).

	Gamma				Erlang				Exponential		
DIC	5075				5071				6075		
	k	a	b	c	k	a	b	c	a	b	c
Mean	19.52	0.449	0.00403	0.0381		0.450	0.00403	0.0381	0.025	0.00424	0.0020
Mode	19.00	0.450	0.00405	0.0370	19	0.450	0.00405	0.0370	0.025	0.00425	0.0020
SD	1.26	0.034	0.00013	0.0025	1	0.035	0.00013	0.0026	0.005	0.00058	0.0002
q-2.5%	17.18	0.380	0.00379	0.0334	18	0.387	0.00378	0.0338	0.017	0.00317	0.0016
q-25%	18.63	0.427	0.00394	0.0362	19	0.426	0.00394	0.0364	0.021	0.00386	0.0019
Median	19.5	0.448	0.00402	0.0380	20	0.448	0.00403	0.0379	0.024	0.00419	0.0020
q-75%	20.38	0.471	0.00412	0.0397	20	0.473	0.00411	0.0396	0.027	0.00462	0.0021
q-97.5%	21.99	0.520	0.00427	0.0432	22	0.528	0.00427	0.0439	0.037	0.00553	0.0024
Confidence range (*)	4.81	0.140	0.00048	0.0098	4	0.141	0.00049	0.0101	0.020	0.00236	0.0008

(*) Confidence range = q-97.5 - q-2.5%.

### Simulation of the Spread of Infection and Disease in the Host Population

**Figure 7:**
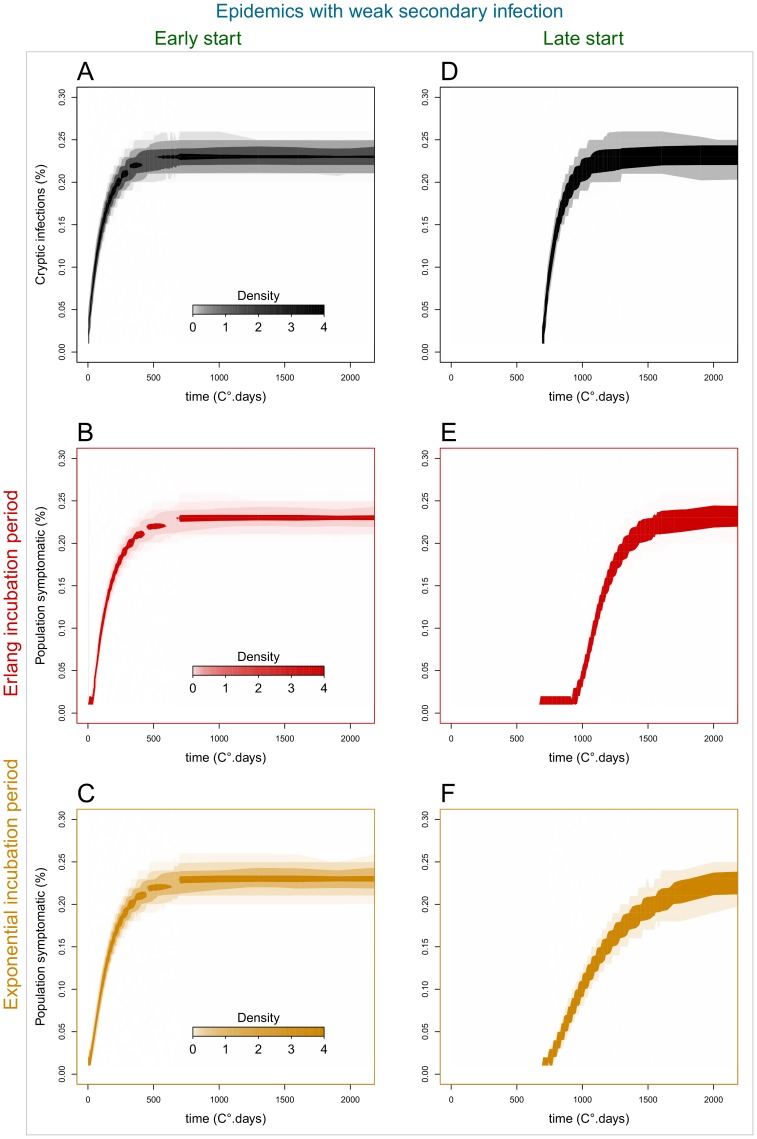
Epidemic dynamics of cryptic infections and disease symptoms in populations with differing incubation period distributions (weak secondary infection). Epidemic dynamics of cryptic infections (top, black) and above-ground disease symptoms in populations with differing incubation period distributions: Erlang (middle, red) and exponential (bottom, orange). Scenarios with low secondary infection rate (β_s_ = 5e-7), and with early (t_0_ = 0°.days) or late (t_0_ = 700°.days) epidemic start in relation to the time of plant sowing, after which resident primary inoculum declines and the incubation period distribution of the aged hosts changes. Each panel: 1000 stochastic simulations of model (5) on a host population in 100*100 square lattice, with α_1_ = 0.002 and α_2_ = 0.008. The shading density represents the proportion of 1000 simulations associated with each point on the graph.

**Figure 8:**
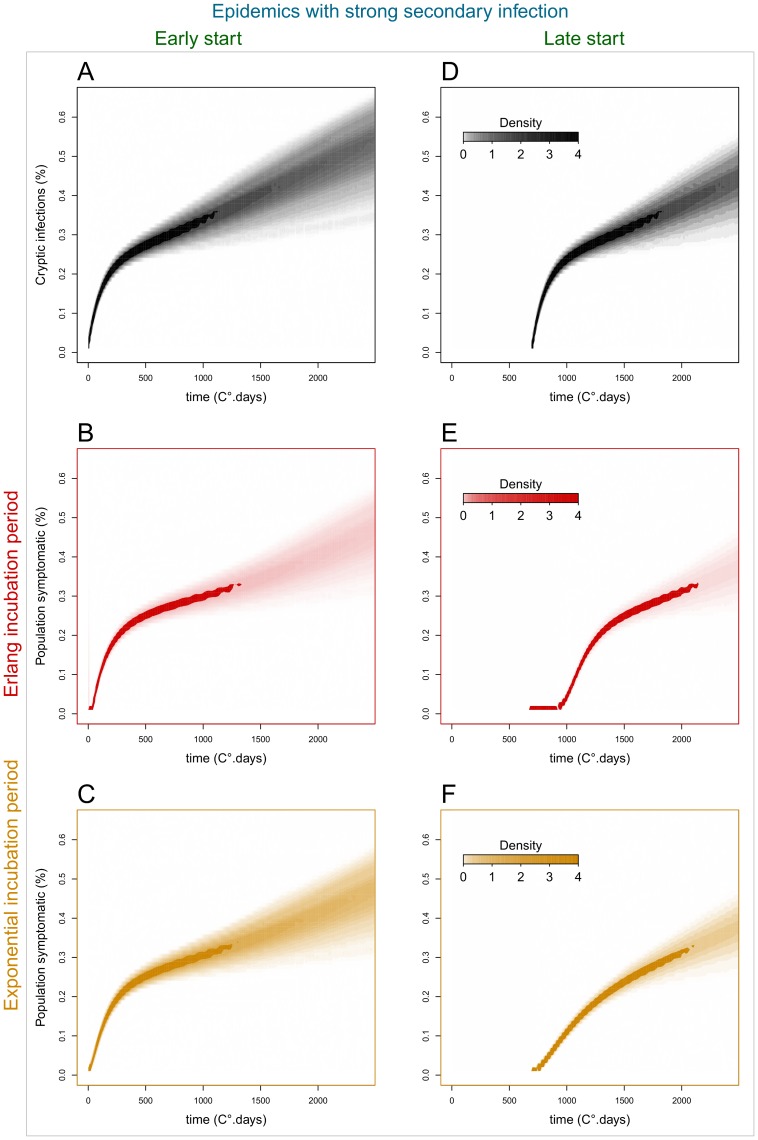
Epidemic dynamics of cryptic infections and disease symptoms in populations with differing incubation period distributions (strong secondary infection). Epidemic dynamics of cryptic infections (top, black) and above-ground disease symptoms in populations with differing incubation period distributions: Erlang (middle, red) and exponential (bottom, orange). Similar to Figure 7, but with a high rate of secondary infection (β_s_ = 5e−4).

**Figure 9:**
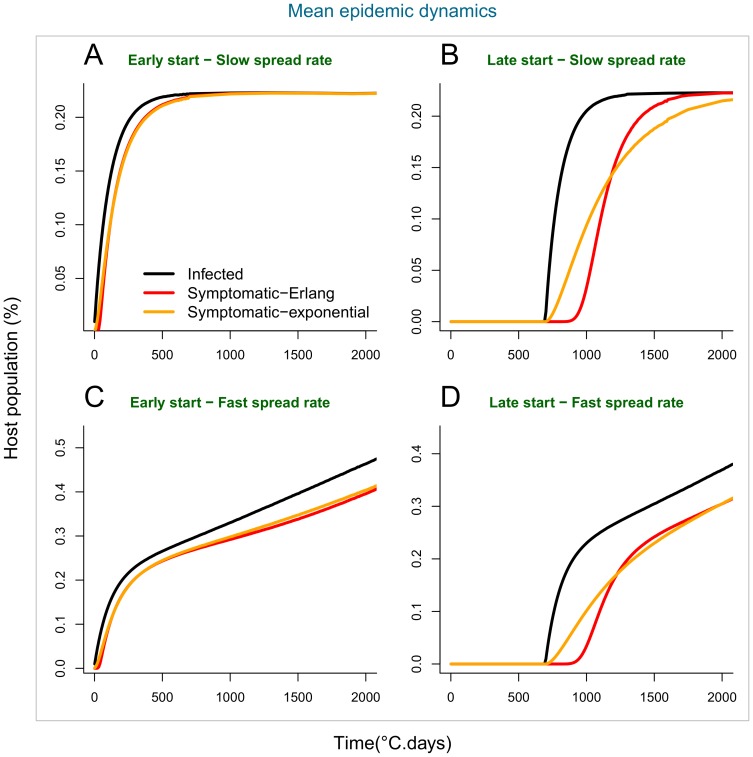
Mean epidemic dynamics of cryptic infections and disease symptoms in populations. Mean epidemic dynamics of cryptic infections (black) and above-ground disease symptoms in populations with differing incubation period distributions: Erlang (red) and exponential (orange). A-B-C & D compare the mean of the distribution of stochastic epidemics at each time point in Figures 7 and 8. Late epidemics show a considerable lag in symptomatic infections relative to cryptic infections that depends on the assumed distribution of the incubation period.

Our simulated scenarios demonstrate there can be considerable differences among the epidemic dynamics of cryptic (asymptomatic) infections and of observable disease (Fig. 7, 8 & 9), depending on the assumed distribution of the incubation period. Epidemics with low secondary infection rate (Fig. 7, 9A & 9B) are driven by declining primary inoculum and exhibit low stochastic variability, while epidemics with high secondary infection rate (Fig. 8, 9C & 9D) show an additional phase of pathogen spread through the host population and greater variability among stochastic realisations. In the first case, cryptic and symptomatic epidemics reach the same asymptote within the crop season (Fig. 9A & 9B), while in the latter case, where the pathogen spreads locally between hosts, the symptomatic epidemic lags after the cryptic epidemic until the end of the crop season (Fig. 9C & 9D).

Differences in the time of epidemic start (t_0_) relative to the time of crop sowing, driven, for example, by environmental conditions or susceptibility of crop variety, further increase the time lag of the observable epidemics. In early epidemics (t_0_ = 0°C.days) (Fig. 7A–C, 8 A–C, 9A & 9C), there is no significant difference between the progress of cryptic and symptomatic infections, and, therefore, a negligible effect of the assumption made about the incubation period distribution, i.e. Erlang or exponential. This pattern is explained by the shortness of the incubation period in young hosts and the resemblance among fitted distribution models prior to 500°C.days (Fig. 5A & 6A). In late epidemics (t_0_ = 700°C.days) there is a significant delay of the symptomatic relative to the cryptic epidemics. This delay is magnified when the incubation period has the more realistic (better data fit) Erlang distribution model (Fig. 7D–E, 8 D–E, 9D & 9E). When the epidemics start late, and, therefore, hosts are older, the mean incubation period is longer and the fitted incubation period distribution models are distinct enough to produce contrasting dynamics between cryptic infection and observable disease. The Erlang (S-(k)I-D) distribution model (5), which fits the observation better, yields a later and more abrupt increase in the number of symptomatic plants than the simplest exponential (S-I-D) model (4) (Fig. 7E–F, 8E–F, 9A & 9D).

## Discussion

Empirical data on the incubation period of pathogens, and in particular soilborne plant pathogen, is rarely available [26]. We have presented the results of detailed experimental observations of the incubation period of the soilborne fungal pathogen *R. solani* in sugar beet, and fitted different probability density functions to the distribution of incubation periods among individual plants. We found that the mode and dispersion of this distribution increase with host age, while a decreasing trend with host age has been observed for some aerial plant diseases [47]. We also found that the single-parameter exponential distribution, which has mode at incubation period zero and is subsumed in the standard epidemiological compartmental-modelling frameworks, fits the incubation data poorly (Table 2, Table 3, Fig. 6). However, the Erlang distribution, which is built into more recent compartmental-modelling frameworks developed in epidemiology in the past two decades [27], [28], [29], [31], provides a data fit as good as other two-parameter distributions such as Weibull (Appendix S2, Table 2, and Table 3). As a further improvement, more elaborate age-varying distribution models could be fitted to the data, particularly when detailed study of specific pathosystems is relevant. For instance, age-varying distribution models with multiple modes (e.g. mixtures of uni-modal distributions) are likely to outperform the statistical fit of our simpler age-varying Erlang model, and, potentially lead to improved prediction of levels of asymptomatic and symptomatic infection in epidemiological models. However, we believe our parsimonious distribution model, based on an Erlang distribution with age-varying rate parameter, provides a plausible and easily-interpretable description of the incubation period of the *R. solani*–sugar beet patho-system that could be used by practitioners to enhance the design of strategies for prevention of root rot disease in sugar beet growing areas.

Furthermore, we have demonstrated, using a spatially-explicit epidemiological model, that the development of observable disease epidemics can lag cryptic pathogen spread significantly (Fig. 7, 8 & 9). Such a lag can mislead crop practitioners that would observe early disease in the field about the extent of infection and risk of further disease, and cause inappropriate decision-making on actions meant to mitigate disease development and economic loss. It is important, therefore, that practitioners have quantitative data about specific pathogen-crop incubation periods and, possibly, alternative monitoring tools that would allow earlier pathogen detection in a crop season. Citrus Huanglongbing is an example of an aerial vector-borne disease with a very long incubation period, where acquisition of knowledge about the incubation period has been central to understanding the severe limitations to managing this disease [48]. Likewise, it is important that epidemiological models for predicting the risk of pathogen spread and disease and the effectiveness of management strategies are parameterised using appropriate incubation period assumptions and data [21]. For example, with the simpler compartments models that assume exponential incubation period distributions, we found that the estimated lag between disease emergence and cryptic infection spread is erroneously reduced. From a disease management perspective, errors in model prediction such as this induced by the use of unsuitable incubation period distributions, could lead to inappropriate decision making regarding application of chemical or biological treatments for preventing crop invasion by pathogens that spread cryptically.

Individual-level data on incubation periods are rarely available for plant, as well as human and animal diseases. The lack of data is often due to difficulty in experimentation, but has additional ethical constraints in the case human and animal diseases. For plant diseases, the development of new monitoring tools such as remote sensing or nuclear magnetic resonance [49], [50] show promise in improving the monitoring of diseases in perennial and non-perennial crop systems, and, therefore, in helping practitioners and epidemiologists to detect pathogen infections and measure incubation periods.

Our epidemiological model is an instance of a hierarchical population dynamics system with hidden (unobserved) states parameterised via individual-level observations. Therefore, a combined empirical and modelling approach like the one we have adopted could help to investigate the role of hidden states and relationships in other population dynamics systems; for example, hidden competition unbalances in communities affected by disease [2], [51] or hidden movement and behaviour of animals [52], [53].

Empirical data on the incubation period of plant-pathogen systems could also be used to facilitate and refine the inference of epidemiological parameters (e.g. rates of infection), that are commonly hidden, from disease data sets. Nowadays, the study of infectious diseases often involves the use of mechanistic-statistical frameworks that incorporate a theoretical-mechanistic population model and a statistical model of the observation process [30], [54]. Recent advances in stochastic integration methods allow epidemiologists to estimate the parameters of stochastic continuous-time models from censored, discrete and incomplete observations of symptomatic individuals among the susceptible population, using, for instance, Bayesian Markov chain Monte Carlo inference methods with data-augmentation and reversible-jump [30], [55], [56]. In this context, incubation period distributions, fitted from empirical data, could be included into the statistical model of the observation process. Albeit, uncertainty on incubation period distributions may remain (especially if it exhibits time-specificity), the introduction of such semi-empirical prior information is likely to improve the numerical integration of the model and refine the estimation of parameters associated with hidden (e.g. latent) states. Regarding the spread *R. solani* in sugar beet, a patho-system for which epidemiological parameters have already been estimated from experimental data, but, with no empirical knowledge on the incubation period [26], it would be very interesting to see how the introduction of more realistic incubation period distributions could affect the estimated parameters.

Finally, our approach combining experimentation and modelling may be difficult to apply to patho-systems that involve, for example, perennial host crops (e.g. the spread of the fungal soilborne pathogens *Rigidoporus lignosus* and *Rhellinus noxius* on rubber trees [7], [57]). In these cases, experimental measurements may be difficult, but it may be doable to i) include incubation periods and ii) estimate their distributions directly from observations of symptomatic individuals. As incubation period distributions are likely to exhibit some kind of time-(or age-) specificity, it may be required to use and compare different semi-empirical functions to capture changes in the mean incubation period with host age or environmental variables.

## Supporting Information

Appendix S1 Experimental data.
(PDF)

Appendix S2 Distribution analysis.
(PDF)

Appendix S3 Age-varying models for the incubation period distribution.
(PDF)
