# Supplementary material for: Estimating the Delay between Host Infection and Disease (Incubation Period) and Assessing Its Significance to the Epidemiology of Plant Diseases
Source: PLoS One. 2014 Jan 22;9(1):e86568. doi: 10.1371/journal.pone.0086568 (PMC3899291; doi:10.1371/journal.pone.0086568)
Supplement: Appendix S1 — Experimental data. (PDF) [file pone.0086568.s001.pdf]

incubation period (days)	age (days)	incubation period (°C days)	age (°C days)
3	18	35,85	182,35
6	18	72,45	182,35
6	18	72,45	182,35
6	18	72,45	182,35
6	18	72,45	182,35
6	18	72,45	182,35
6	18	72,45	182,35
6	18	72,45	182,35
6	18	72,45	182,35
6	18	72,45	182,35
6	18	72,45	182,35
6	18	72,45	182,35
6	18	72,45	182,35
6	18	72,45	182,35
6	18	72,45	182,35
6	18	72,45	182,35
6	18	72,45	182,35
6	18	72,45	182,35
6	18	72,45	182,35
6	18	72,45	182,35
6	18	72,45	182,35
6	18	72,45	182,35
6	18	72,45	182,35
6	18	72,45	182,35
6	18	72,45	182,35
6	18	72,45	182,35
6	18	72,45	182,35
6	18	72,45	182,35
6	18	72,45	182,35
6	18	72,45	182,35
6	18	72,45	182,35
6	18	72,45	182,35
6	18	72,45	182,35
6	18	72,45	182,35
6	18	72,45	182,35
6	18	72,45	182,35
6	18	72,45	182,35
6	18	72,45	182,35
6	18	72,45	182,35
6	18	72,45	182,35
6	18	72,45	182,35
6	18	72,45	182,35
6	18	72,45	182,35
6	18	72,45	182,35
6	18	72,45	182,35
6	18	72,45	182,35
7	18	85,5	182,35
7	18	85,5	182,35
7	18	85,5	182,35

7	18	85,5	182,35
7	18	85,5	182,35
7	18	85,5	182,35
7	18	85,5	182,35
7	18	85,5	182,35
7	18	85,5	182,35
7	18	85,5	182,35
7	18	85,5	182,35
7	18	85,5	182,35
7	18	85,5	182,35
7	18	85,5	182,35
7	18	85,5	182,35
7	18	85,5	182,35
7	18	85,5	182,35
7	18	85,5	182,35
7	18	85,5	182,35
7	18	85,5	182,35
7	18	85,5	182,35
7	18	85,5	182,35
7	18	85,5	182,35
8	18	98,65	182,35
8	18	98,65	182,35
8	18	98,65	182,35
8	18	98,65	182,35
8	18	98,65	182,35
8	18	98,65	182,35
8	18	98,65	182,35
8	18	98,65	182,35
10	18	124,95	182,35
4	32	50,85	359,25
4	32	50,85	359,25
8	32	101,45	359,25
8	32	101,45	359,25
8	32	101,45	359,25
8	32	101,45	359,25
8	32	101,45	359,25
8	32	101,45	359,25
8	32	101,45	359,25
8	32	101,45	359,25
8	32	101,45	359,25
8	32	101,45	359,25
8	32	101,45	359,25
8	32	101,45	359,25
8	32	101,45	359,25
8	32	101,45	359,25
8	32	101,45	359,25
8	32	101,45	359,25
9	32	115,3	359,25
9	32	115,3	359,25
9	32	115,3	359,25

9	32	115,3	359,25
9	32	115,3	359,25
9	32	115,3	359,25
9	32	115,3	359,25
10	32	128,85	359,25
10	32	128,85	359,25
10	32	128,85	359,25
10	32	128,85	359,25
10	32	128,85	359,25
10	32	128,85	359,25
12	32	156,1	359,25
12	32	156,1	359,25
12	32	156,1	359,25
12	32	156,1	359,25
12	32	156,1	359,25
12	32	156,1	359,25
12	32	156,1	359,25
12	32	156,1	359,25
12	32	156,1	359,25
12	32	156,1	359,25
12	32	156,1	359,25
12	32	156,1	359,25
12	32	156,1	359,25
12	32	156,1	359,25
12	32	156,1	359,25
13	32	169,8	359,25
13	32	169,8	359,25
13	32	169,8	359,25
13	32	169,8	359,25
14	32	183,9	359,25
14	32	183,9	359,25
9	46	121,2	542
9	46	121,2	542
9	46	121,2	542
9	46	121,2	542
9	46	121,2	542
10	46	135,7	542
10	46	135,7	542
12	46	166,35	542
12	46	166,35	542
12	46	166,35	542
12	46	166,35	542
12	46	166,35	542
12	46	166,35	542
12	46	166,35	542
14	46	194,65	542
14	46	194,65	542
14	46	194,65	542
14	46	194,65	542
14	46	194,65	542

14	46	194,65	542
14	46	194,65	542
14	46	194,65	542
14	46	194,65	542
14	46	194,65	542
14	46	194,65	542
14	46	194,65	542
14	46	194,65	542
14	46	194,65	542
14	46	194,65	542
14	46	194,65	542
14	46	194,65	542
14	46	194,65	542
14	46	194,65	542
14	46	194,65	542
14	46	194,65	542
15	46	208,45	542
15	46	208,45	542
15	46	208,45	542
15	46	208,45	542
15	46	208,45	542
16	46	222,5	542
16	46	222,5	542
19	46	262,35	542
19	46	262,35	542
19	46	262,35	542
19	46	262,35	542
19	46	262,35	542
19	46	262,35	542
19	46	262,35	542
19	46	262,35	542
19	46	262,35	542
20	46	276,25	542
20	46	276,25	542
14	60	204,1	607,05
14	60	204,1	607,05
14	60	204,1	607,05
17	60	252,6	607,05
17	60	252,6	607,05
17	60	252,6	607,05
17	60	252,6	607,05
17	60	252,6	607,05
17	60	252,6	607,05
17	60	252,6	607,05
17	60	252,6	607,05
17	60	252,6	607,05
17	60	252,6	607,05
17	60	252,6	607,05
17	60	252,6	607,05
17	60	252,6	607,05

17	60	252,6	607,05
17	60	252,6	607,05
17	60	252,6	607,05
17	60	252,6	607,05
17	60	252,6	607,05
17	60	252,6	607,05
20	60	304,85	607,05
20	60	304,85	607,05
20	60	304,85	607,05
20	60	304,85	607,05
20	60	304,85	607,05
20	60	304,85	607,05
20	60	304,85	607,05
20	60	304,85	607,05
20	60	304,85	607,05
20	60	304,85	607,05
20	60	304,85	607,05
20	60	304,85	607,05
20	60	304,85	607,05
20	60	304,85	607,05
20	60	304,85	607,05
20	60	304,85	607,05
20	60	304,85	607,05
20	60	304,85	607,05
20	60	304,85	607,05
20	60	304,85	607,05
20	60	304,85	607,05
20	60	304,85	607,05
20	60	304,85	607,05
20	60	304,85	607,05
20	60	304,85	607,05
20	60	304,85	607,05
23	60	358,6	607,05
14	74	242,8	811,15
14	74	242,8	811,15
16	74	278,45	811,15
16	74	278,45	811,15
16	74	278,45	811,15
16	74	278,45	811,15
16	74	278,45	811,15
16	74	278,45	811,15
16	74	278,45	811,15
16	74	278,45	811,15
16	74	278,45	811,15
20	74	352,9	811,15
20	74	352,9	811,15
20	74	352,9	811,15
20	74	352,9	811,15
20	74	352,9	811,15
20	74	352,9	811,15

20	74	352,9	811,15
20	74	352,9	811,15
20	74	352,9	811,15
20	74	352,9	811,15
20	74	352,9	811,15
20	74	352,9	811,15
20	74	352,9	811,15
21	74	371,05	811,15
21	74	371,05	811,15
21	74	371,05	811,15
21	74	371,05	811,15
21	74	371,05	811,15
21	74	371,05	811,15
21	74	371,05	811,15
21	74	371,05	811,15
21	74	371,05	811,15
21	74	371,05	811,15
21	74	371,05	811,15
21	74	371,05	811,15
28	74	492,2	811,15
28	74	492,2	811,15
28	74	492,2	811,15
28	74	492,2	811,15
28	74	492,2	811,15
28	74	492,2	811,15
28	74	492,2	811,15
31	74	543,95	811,15
31	74	543,95	811,15
31	74	543,95	811,15
17	88	301,15	1053,95
17	88	301,15	1053,95
17	88	301,15	1053,95
17	88	301,15	1053,95
17	88	301,15	1053,95
17	88	301,15	1053,95
17	88	301,15	1053,95
17	88	301,15	1053,95
17	88	301,15	1053,95
22	88	388,1	1053,95
22	88	388,1	1053,95
22	88	388,1	1053,95
23	88	405,95	1053,95
23	88	405,95	1053,95
23	88	405,95	1053,95
23	88	405,95	1053,95
23	88	405,95	1053,95
23	88	405,95	1053,95
23	88	405,95	1053,95
23	88	405,95	1053,95
28	88	491,05	1053,95

28	88	491,05	1053,95
28	88	491,05	1053,95
28	88	491,05	1053,95
28	88	491,05	1053,95
28	88	491,05	1053,95
28	88	491,05	1053,95
28	88	491,05	1053,95
28	88	491,05	1053,95
28	88	491,05	1053,95
28	88	491,05	1053,95
28	88	491,05	1053,95
28	88	491,05	1053,95
28	88	491,05	1053,95
28	88	491,05	1053,95
28	88	491,05	1053,95
28	88	491,05	1053,95
28	88	491,05	1053,95
28	88	491,05	1053,95
28	88	491,05	1053,95
28	88	491,05	1053,95
28	88	491,05	1053,95
30	88	523,7	1053,95
30	88	523,7	1053,95
30	88	523,7	1053,95
30	88	523,7	1053,95
30	88	523,7	1053,95
38	88	652,15	1053,95
38	88	652,15	1053,95
9	102	156,55	1303,35
14	102	241,65	1303,35
14	102	241,65	1303,35
14	102	241,65	1303,35
14	102	241,65	1303,35
24	102	402,75	1303,35
24	102	402,75	1303,35
24	102	402,75	1303,35
24	102	402,75	1303,35
24	102	402,75	1303,35
24	102	402,75	1303,35
24	102	402,75	1303,35
24	102	402,75	1303,35
24	102	402,75	1303,35
24	102	402,75	1303,35
29	102	476,5	1303,35
29	102	476,5	1303,35
29	102	476,5	1303,35
29	102	476,5	1303,35
29	102	476,5	1303,35
29	102	476,5	1303,35
29	102	476,5	1303,35

29	102	476,5	1303,35
29	102	476,5	1303,35
29	102	476,5	1303,35
29	102	476,5	1303,35
29	102	476,5	1303,35
29	102	476,5	1303,35
29	102	476,5	1303,35
29	102	476,5	1303,35
29	102	476,5	1303,35
29	102	476,5	1303,35
29	102	476,5	1303,35
29	102	476,5	1303,35
29	102	476,5	1303,35
31	102	506,8	1303,35
31	102	506,8	1303,35
31	102	506,8	1303,35
31	102	506,8	1303,35
31	102	506,8	1303,35
31	102	506,8	1303,35
31	102	506,8	1303,35
31	102	506,8	1303,35
36	102	587,2	1303,35
36	102	587,2	1303,35
15	116	234,85	1545
17	116	265,15	1545
17	116	265,15	1545
17	116	265,15	1545
17	116	265,15	1545
17	116	265,15	1545
17	116	265,15	1545
17	116	265,15	1545
22	116	345,55	1545
22	116	345,55	1545
22	116	345,55	1545
22	116	345,55	1545
22	116	345,55	1545
28	116	435,25	1545
28	116	435,25	1545
28	116	435,25	1545
28	116	435,25	1545
28	116	435,25	1545
31	116	478,1	1545
31	116	478,1	1545
31	116	478,1	1545
31	116	478,1	1545
31	116	478,1	1545
31	116	478,1	1545
31	116	478,1	1545
34	116	523	1545
34	116	523	1545

34	116	523	1545
38	116	580,9	1545
38	116	580,9	1545
38	116	580,9	1545
38	116	580,9	1545
38	116	580,9	1545
38	116	580,9	1545
38	116	580,9	1545
42	116	639	1545
42	116	639	1545
42	116	639	1545
42	116	639	1545
42	116	639	1545
42	116	639	1545
42	116	639	1545
45	116	680,9	1545
51	116	755,8	1545
51	116	755,8	1545
51	116	755,8	1545
24	130	361,05	1764,85
24	130	361,05	1764,85
24	130	361,05	1764,85
24	130	361,05	1764,85
28	130	419,15	1764,85
28	130	419,15	1764,85
28	130	419,15	1764,85
28	130	419,15	1764,85
28	130	419,15	1764,85
31	130	461,05	1764,85
31	130	461,05	1764,85
31	130	461,05	1764,85
31	130	461,05	1764,85
31	130	461,05	1764,85
31	130	461,05	1764,85
31	130	461,05	1764,85
31	130	461,05	1764,85
31	130	461,05	1764,85
31	130	461,05	1764,85
31	130	461,05	1764,85
34	130	497,75	1764,85
34	130	497,75	1764,85
34	130	497,75	1764,85
34	130	497,75	1764,85
34	130	497,75	1764,85
37	130	535,95	1764,85
37	130	535,95	1764,85
37	130	535,95	1764,85
37	130	535,95	1764,85
37	130	535,95	1764,85
37	130	535,95	1764,85

37	130	535,95	1764,85
37	130	535,95	1764,85
37	130	535,95	1764,85
43	130	604,7	1764,85
43	130	604,7	1764,85
43	130	604,7	1764,85
55	130	756,5	1764,85
55	130	756,5	1764,85
55	130	756,5	1764,85
55	130	756,5	1764,85
57	130	779,8	1764,85
57	130	779,8	1764,85
59	130	802,05	1764,85
59	130	802,05	1764,85
59	130	802,05	1764,85
