# Supplementary material for: Estimating the Delay between Host Infection and Disease (Incubation Period) and Assessing Its Significance to the Epidemiology of Plant Diseases
Source: PLoS One. 2014 Jan 22;9(1):e86568. doi: 10.1371/journal.pone.0086568 (PMC3899291; doi:10.1371/journal.pone.0086568)
Supplement: Appendix S2 — Distribution analysis. (PDF) [file pone.0086568.s002.pdf]

Appendix S2 : Distribution Analysis

1. Probability distributions

1.1. *Gamma, Erlang and exponential distributions*

- The Gamma distribution is a two-parameter continuous-variable probability distribution with density function:

$$f(x,k,\lambda)=\frac{\lambda^k x^{k-1}}{\Gamma(k)}e^{-\lambda x} \qquad \text{(s.1)}$$

For x ≥0, k>0 (shape parameter), and λ>0 (rate parameter), and where Γ() is the gamma function

- The Erlang distribution is a particular case of the Gamma distribution when the shape parameter k is an integer. The *pdf* of the Erlang distribution is:

$$f(x,k,\lambda)=\frac{\lambda^k x^{k-1}}{(k-1)!}e^{-\lambda x} \qquad \text{(s.2)}$$

For x ≥0, λ>0, and k a positive integer.

- The exponential distribution is an Erlang distribution with shape parameter k=1; it has *pdf*:

$$f(x,\lambda)=\lambda e^{-\lambda x} \qquad \text{(s.3)}$$

1.2. *Weibull distribution*

The Weibull distribution is a two-parameter continuous-variable probability distribution with *pdf*:

$$f(x,k,\lambda)=\lambda k\left(\lambda x\right)^{k-1}e^{-(\lambda x)^k} \qquad \text{(s.4)}$$

For x ≥0, k>0 (shape parameter) and λ >0 (rate parameter).

1.3. *Lognormal distribution*

The Lognormal distribution is a two-parameter probability distribution of a continuous random variable whose logarithm is normally distributed. The *pdf* of a log-normal distribution is:

$$f(x,\mu,\sigma)=\frac{1}{x\sigma\sqrt{2\pi}}e^{-\frac{(\ln(x)-\mu)^2}{2\sigma^2}} \qquad \text{(s.5)}$$

For x ≥0, and where μ and σ are the mean and standard deviation of the logarithm of x.

2. Age-by-age fitted probability density functions (*pdf*): Figure S.1

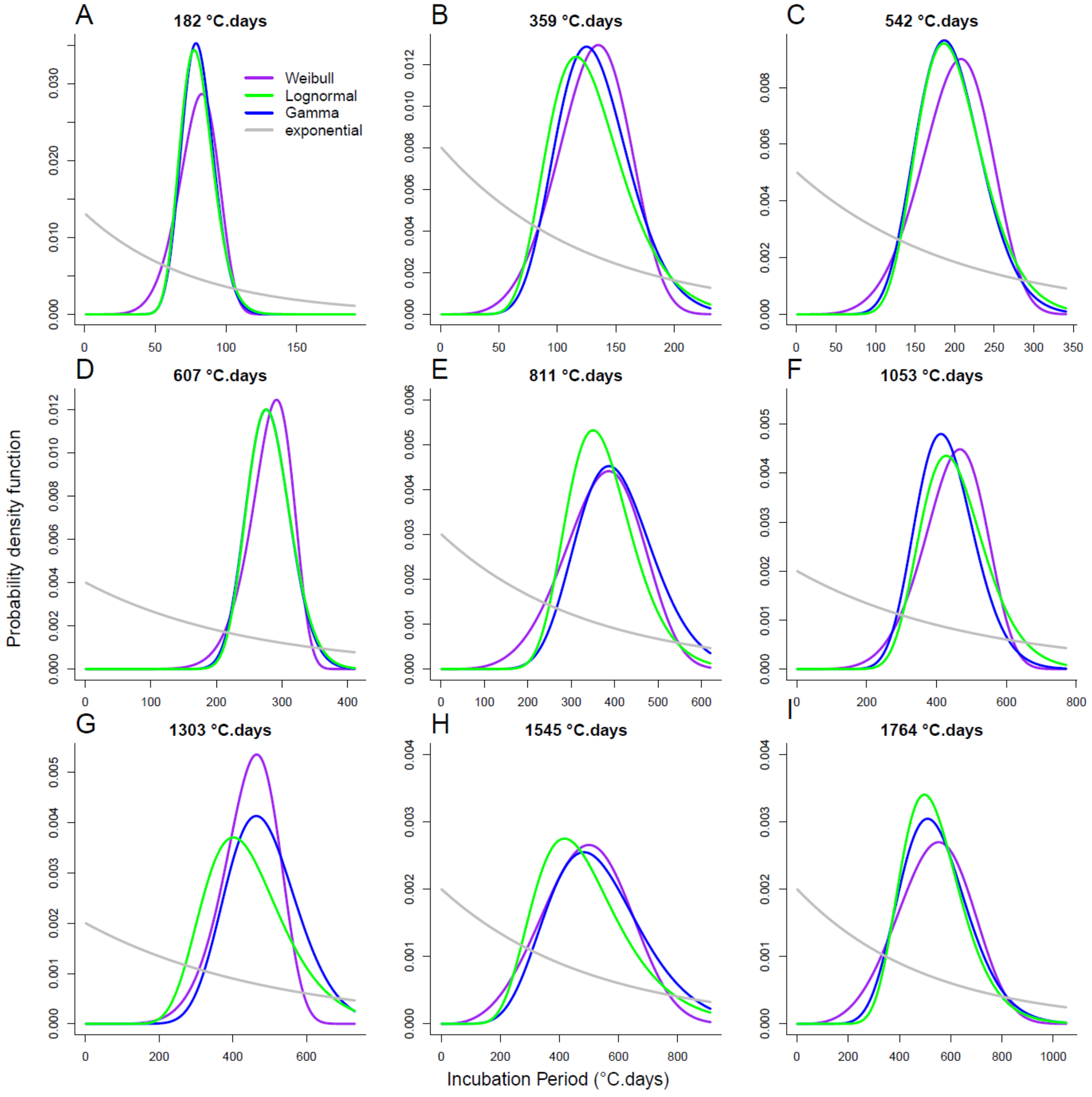

Figure S.1 Probability density functions of the distribution models fitted to the incubation period data by host age: Gamma (blue line) Lognormal (green), Weibull (purple) and exponential (grey). Age of host plants at inoculation: (A) 18 days (182 °C.days), (B) 32 days (359 °C.days), (C) 46 days (542 °C.days), (D) 60 days (607 °C.days), (E) 74 days (811 °C.days), (F) 88 days (1053 °C.days), (G) 102 days (1303 °C.days), (H) 116 days (1545 °C.days) ,and (I) 130 days (1764 °C.days).
