# Supplementary material for: Estimating the Delay between Host Infection and Disease (Incubation Period) and Assessing Its Significance to the Epidemiology of Plant Diseases
Source: PLoS One. 2014 Jan 22;9(1):e86568. doi: 10.1371/journal.pone.0086568 (PMC3899291; doi:10.1371/journal.pone.0086568)
Supplement: Appendix S3 — Age-varying models for the incubation period distribution. (PDF) [file pone.0086568.s003.pdf]

Appendix S3: Age-varying models of the incubation period distribution

1. Preliminary study for the rate parameter λ(t): Table S.2

Age-by-age and joint estimations of the parameters Gamma distribution (s.1) (Table 2, Table S.1) suggested that the rate parameter λ is described by an exponential-like decreasing function. Moreover the assumption of a decrease in time of the rate parameter, that allows the mean and variance of the incubation period to increase with time, was consistent with trends observed in raw data (Fig. 3 & Table 1). In order to find a good candidate function for describing how the rate parameter changes over time, with fitted several parametric models and used the Akaike information criterion (AIC) for comparing their goodness-of-fit (Table S.2).

Table S.2: Fitted age-varying models built from Gamma distributions with constant shape parameter k and an age-dependent decreasing rate parameter λ(t): parameters and Akaike information criterion.

**Model 1**	**lambda=a*exp(-b*t^2)**			
**k**	**a**	**b**		**AIC**
4.9	2.9e-02	5.0e-07		5741
**Model 2**	**lambda=a*exp(-b*t^2)+c**			
**k**	**a**	**b**	**c**	**AIC**
19.2	0.23	5.2e-06	4.0e-02	5084
**Model 3**	**lambda=a*exp(-b*t)**			
**k**	**a**	**b**		**AIC**
7.7	7.8e-02	1.2e-03		5511
**Model 4**	**lambda=a*exp(-b*t)+c**			
**k**	**a**	**b**	**c**	**AIC**
19.5	0.48	4.0e-03	3.8e-02	5079
**Model 5**	**lambda=a*exp(-b*t^0,5)**			
**k**	**a**	**b**		**AIC**
11.4	0.29	6.9e-02		5331
**Model 6**	**lambda=a*exp(-b*t^0,5)+c**			
**k**	**a**	**b**	**c**	**AIC**
18.4	1.57	1.5e-01	3.1e-02	5105
**Model 7**	**lambda=a/(1+a*t/b)**			
**k**	**a**	**b**		**AIC**
15.5	1.19	43.2		5184
**Model 8**	**lambda=a/(1+a*t/b)+c**			
**k**	**a**	**b**	**c**	**AIC**
16.7	13.33	37.21	7.6e-03	5149

2. Bayesian inference for the parameters of the age-specific distributions

2.1. Supplementary information for parameters estimation

The probability densities of the parameters k, a, b and c of the age-varying distribution models of the incubation period (i.e. Gamma, Erlang and exponential) were estimated from the experimental data using a Bayesian framework. Posterior densities were obtained by running three Markov Chains with initial values drawn randomly. The convergence of the posterior distribution was assessed by examining the trace, autocorrelation plots, and marginal posterior densities. In order to examine the presence of an undesirable drift in the trace, which can sometimes show after many iterations, we sampled the chain over a very large number of iterations (1 Million). Non-informative prior distributions were obtained from uniform Beta(1,1) distributions for a, b and c ,and a uniform prior for k (when k was a free parameter). Our sensitivity analyses suggested that the upper bounds we used in the uniform prior distributions had no influence on the posterior distributions.

2.2. BUGS base code

Probability densities of the parameters k, a, b, and c can be reproduced by running on OpenBUGS or WinBUGS software the following base code:

```
#model
model{
#priors
#a~dunif(0,100)
#b~dunif(0,100)
#c~dunif(0,100)
k~dunif(0,100)
a~dbeta(1,1)
b~dbeta(1,1)
c~dbeta(1,1)
for(i in 1:N){
lambda[i]<-a*exp(-b*t[i])+c
#likelihood
T[i]~dgamma(k,lambda[i])
}
}
```

2.3. Trace plots, Autocorrelation plots, and posterior densities

- Gamma age-varying distribution

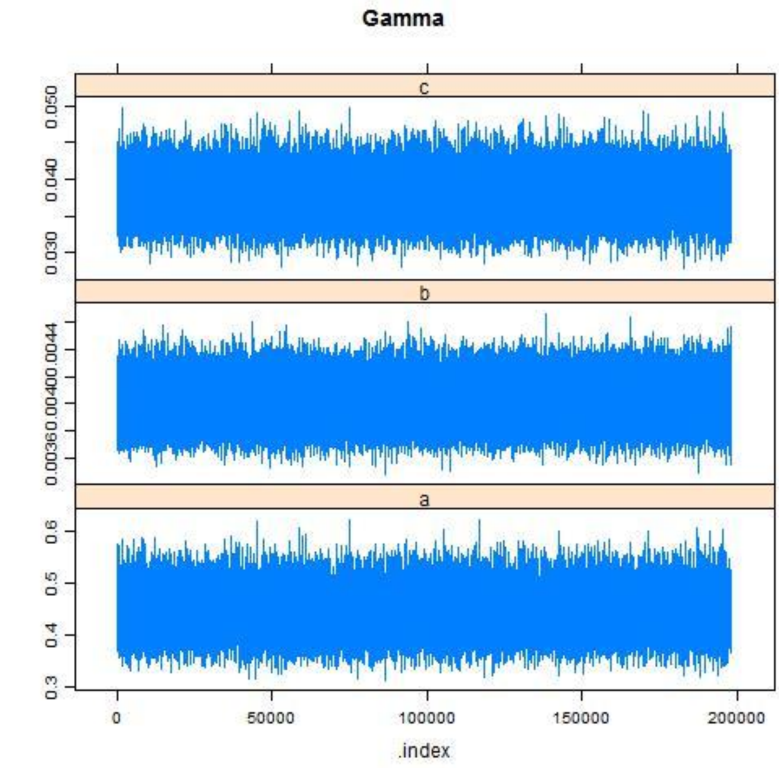

Figure S.2 Trace of parameters a, b, and c in the fitted age-varying Gamma distribution of the incubation period. The plots represent parameter samples versus iteration step in a Markov chain with 1 000 000 of iterations.

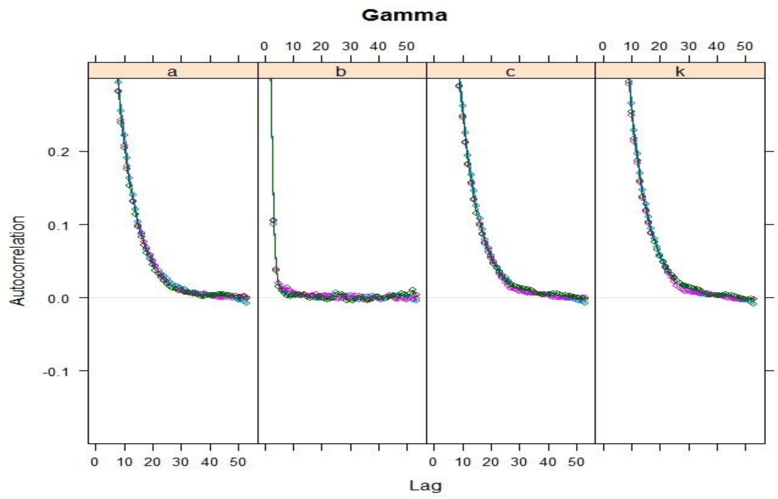

Figure S.3 Autocorrelation of parameters a, b, c and k in the fitted age-varying Gamma distribution of the incubation period.

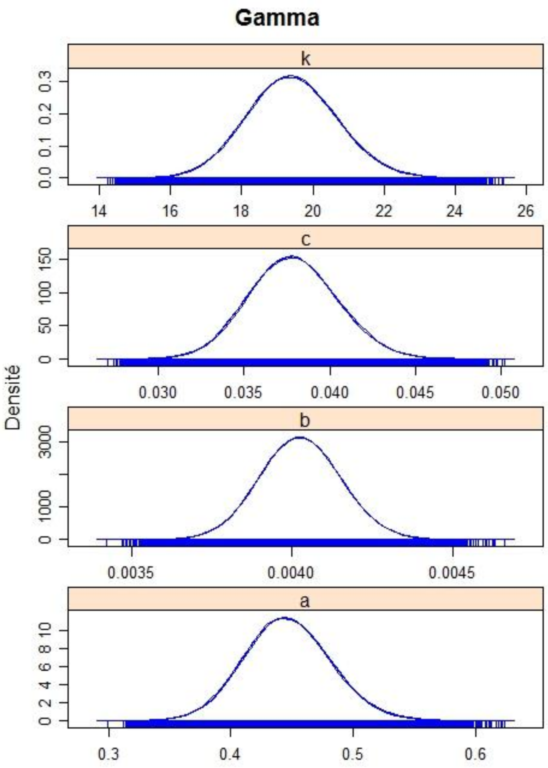

Figure S.4 Marginal posterior density of parameters a, b, c and k in the fitted age-varying Gamma distribution of the incubation period.

- Erlang age-varying distribution

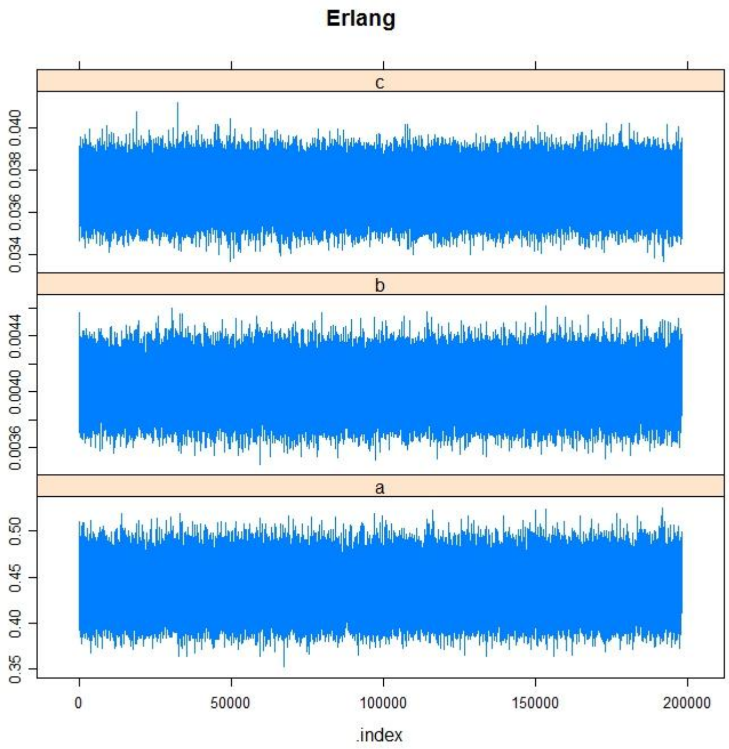

Figure S.5 Trace of parameters a, b and c in the fitted age-varying Erlang distribution of the incubation period. The plots represent parameter samples versus iteration step in a Markov chain with 1 000 000 of iterations.

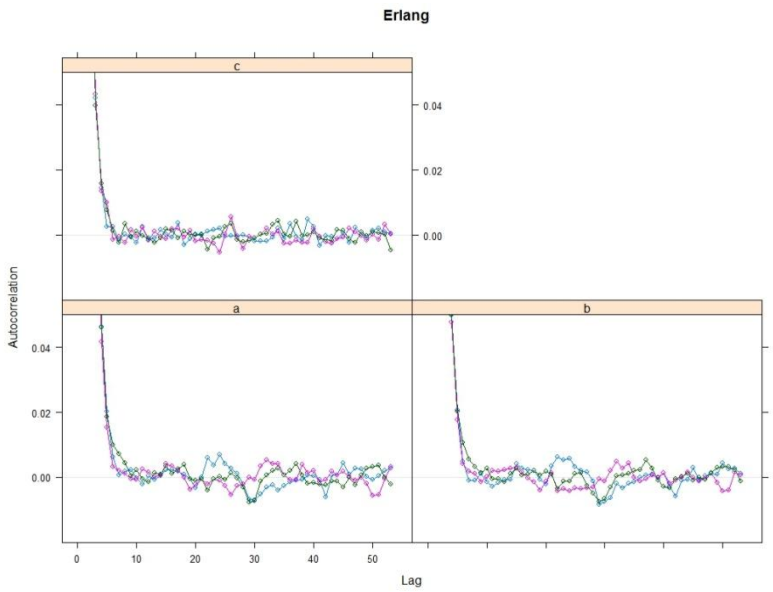

Figure S.6 Autocorrelation of parameters a, b and c in the fitted age-varying Erlang distribution of the incubation period.

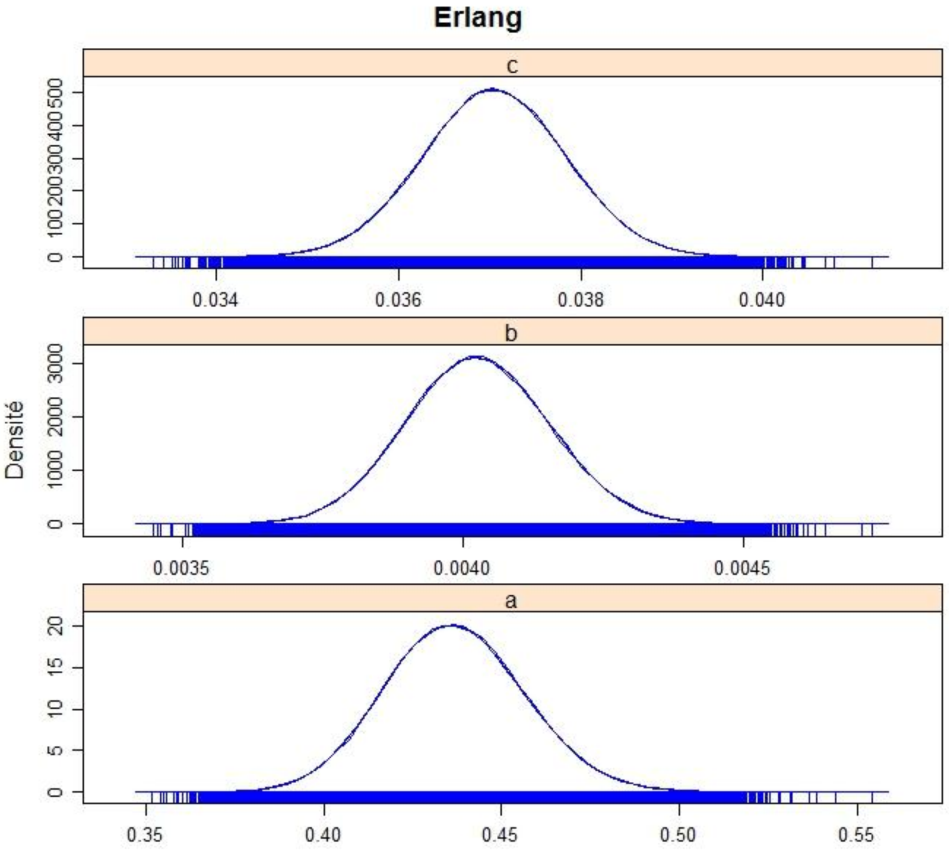

Figure S.7 Marginal posterior density of parameters a, b and c in the fitted age-varying Erlang distribution of theincubation period.

- Exponential age-varying distribution

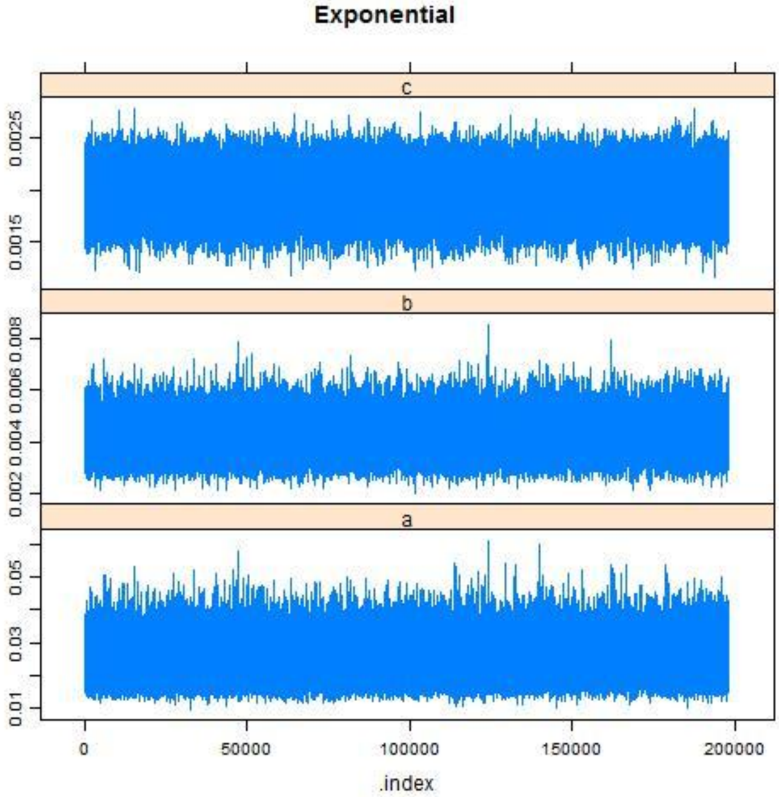

Figure S.8 Trace of parameters a, b and c in the fitted age-varying exponential distribution of the incubation period. The plots represent parameter samples versus iteration step in a Markov chain with 1 000 000 of iterations.

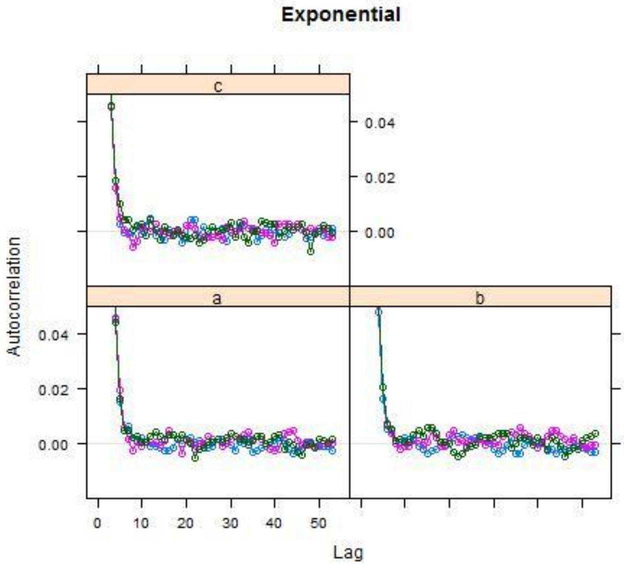

Figure S.9 Autocorrelation of parameters a, b and c in the fitted age-varying exponential distribution of the incubation period.

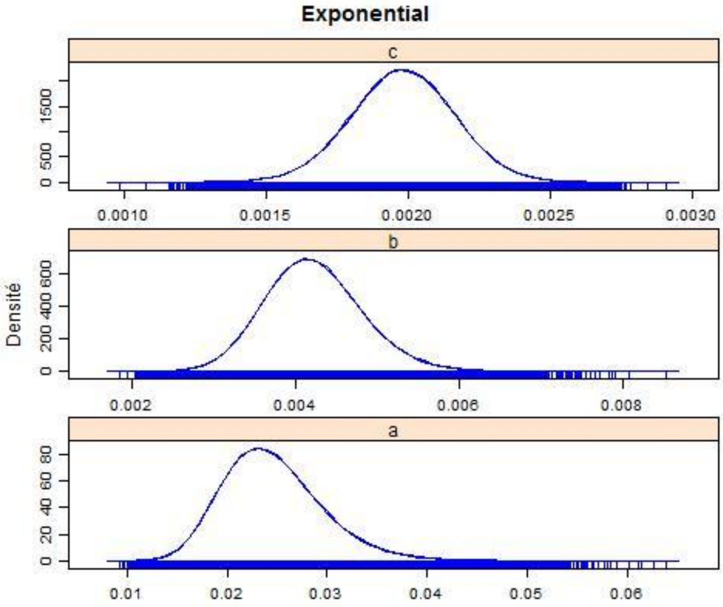

Figure S.10 Marginal posterior density of parameters a, b and c in the fitted age-varying exponential distribution of the incubation period.
